# Public Health Risks Associated with Tetrodotoxin and Its Analogues in European Waters: Recent Advances after The EFSA Scientific Opinion

**DOI:** 10.3390/toxins11050240

**Published:** 2019-04-26

**Authors:** Panagiota Katikou

**Affiliations:** Ministry of Rural Development and Food, Directorate General of Rural Development, Directorate of Research, Innovation and Education, Hapsa & Karatasou 1, 54626 Thessaloniki, Greece; pkatikou@minagric.gr or pkatikou@otenet.gr; Tel.: +30-2310-551617

**Keywords:** tetrodotoxin, tetrodotoxin analogues, health risks, bivalve molluscs, gastropods, European waters, analysis methods, toxicity evaluation, occurrence data, exposure assessment

## Abstract

Tetrodotoxin (TTX) and its analogues are naturally occurring toxins responsible worldwide for human intoxication cases and fatalities, mainly associated with pufferfish consumption. In the last decade, TTXs were detected in marine bivalves and gastropods from European waters. As TTXs are not regulated or monitored at EU level, their unexpected occurrence in shellfish raised concerns as a food safety hazard and revealed the necessity of a thorough assessment on the public health risks associated with their presence. For this reason, the European Food Safety Authority (EFSA) was requested by the European Commission to provide a scientific opinion, finally adopted in March 2017, according to which a provisional concentration below 44 μg TTX equivalents/kg shellfish meat, based on a large portion size of 400 g, was considered not to result in adverse effects in humans. The EFSA expert panel, however, recognized a number of shortcomings and uncertainties related to the unavailability of sufficient scientific data and provided relevant recommendations for future research to overcome these data gaps identified in order to further refine the risk assessment on TTXs. The present review aims to summarize the knowledge obtained towards addressing these recommendations in the two years following publication of the EFSA opinion, at the same time highlighting the points requiring further investigation.

## 1. Introduction

Tetrodotoxin (TTX) is a naturally-occurring neurotoxin known to cause human poisoning incidents, sometimes lethal, and historically related to the consumption of certain pufferfish species, especially in Japan where the so-called “fugu” dish is considered a delicacy [1]. Until 2007, TTX mainly existed in tropical waters while its presence had never been considered as a potential threat in European waters. This was however changed by two separate cases, with their records appearing more or less simultaneously: the establishment of the TTX-containing pufferfish *Lagocephalus sceleratus* in the Mediterranean Sea, after its invasion through the Suez canal [2,3,4], and the first record of human intoxication due to TTX ingestion, caused by a *Charonia lampas lampas* trumpet shell originating from the south Portuguese coasts and purchased in Malaga, Spain [5,6]. Some years later, in 2015, the first evidence on the presence of TTX and its analogues (TTXs) in European bivalve molluscs started to appear in the literature, initially with mussels, pacific oysters and clams harvested in 2013 and 2014 in the UK [7], followed by mussels and venus clams harvested between 2006–2014 in Greece [8], and finally in mussels and oysters harvested in 2015 in the Netherlands [9,10].

The presence of TTXs in different European countries together with the above intoxication case and the known very toxic nature of the compound, raised concerns about TTX as a food safety hazard in Europe, due to the lack of a maximum permitted level (MPL) for TTXs content in seafood within the European Union (EU) and the fact that this toxin group is not monitored on a regular basis [11,12]. Indeed, the only relevant requirement foreseen in the current EU legislative framework is that fishery products derived from poisonous fish of the family Tetraodontidae must not be placed on the market [13,14]. As a result, national health measures were introduced in the Netherlands [10] and subsequently the European Commission requested the “EFSA Panel on Contaminants in the Food Chain” to deliver a scientific opinion on the risks related to the presence of TTXs in marine bivalves and gastropods [15]; this opinion was adopted in March 2017 and published later in April [16].

Within the EFSA scientific opinion, the CONTAM Panel analysis of data available at the time the risk assessment was performed resulted in the introduction of a proposed safe concentration of lower than 44 μg TTX eq/kg of shellfish meat, which was considered as not expected to result in adverse effects in humans (NOAEL), with calculations based on a large portion size (400 g), an adult body weight of 70 kg and a group acute reference dose (ARfD) of 0.25 μg/kg b.w. On the other hand, the opinion identified a series of uncertainty factors and several limitations due to the inadequacy of relevant data, which resulted in a number of recommendations for necessary information, required in order to provide a more refined exposure assessment. For this purpose, the availability of further information and more data was considered critical with respect to the following: (a) occurrence of TTXs in edible parts of marine bivalves and gastropods from different EU waters; (b) data on TTXs concentrations obtained using EU approved and validated chemical-analytical methods; (c) existence of certified standards and reference materials for TTXs; (d) information on the fate of TTXs during cooking; (e) Studies on the sources and critical factors leading to the accumulation of TTXs in marine bivalves and gastropods; (f) further information on toxicokinetics of TTXs; (g) further information on the acute oral toxicity of TTXs but also investigations on their chronic effects; (h) adequate and well described evidence to estimate the relative potencies of TTX analogues, preferentially after oral exposure and (i) investigation on the possibility to combine both STXs and TTXs in one health-based guidance value (HBGV) due to the fact that these toxins exert similar toxic effects via a similar mode of action [16].

In the two year period after the EFSA opinion was published, relevant studies and information made available by the scientific community have been adding up. Some of the new knowledge established, especially new data on occurrence and acute toxicity indicating a lower risk level, have raised concerns in the shellfish industry, underlining a threat to intra-community trade and a negative effect on the image of shellfish in general, due to the possibility of the TTX case being an unfounded ‘epidemic’ [17]. On the contrary, the recently published game-changing report on TTXs chronic toxicity potential after repeated exposure to low oral doses, including the dose of 75 μg/kg b.w. [18], raises doubts towards the opposite direction regarding calculation of the above EFSA-proposed concentration, as at the time of that risk assessment the aforementioned dose was documented as a NOAEL for acute oral toxicity [19]. In this context, the present review aims to provide a summary of the recent advances concerning the public health risks associated with the presence of TTXs in Europe two years after adoption of the relevant EFSA scientific opinion [16]. Using the same approach and following the conclusions and recommendations of that particular publication, a re-initiation of scientific dialogue is proposed for the refinement of the existing risk assessment and establishment of at least a uniform regulatory management approach for this toxin group at EU level.

## 2. Sources and Origin of TTX

The origin of TTX is thought to be associated to a wide diversity of bacterial species and strains. There are reports in the literature of at least 150 TTX-producing bacterial strains, with representatives of the *Vibrio* genus comprising more than 30% of all TTX-producing strains. More specifically, a connection between toxin production and the presence of *V. alginolyticus* in the aquatic animals’ microflora has been reported. On the other hand, representatives of the genus *Bacillus* comprise approximately 15% of the isolated TTX-producing strains, while *Pseudomonas*, *Aeromonas*, *Alteromonas*, *Streptomyces*, and *Roseobacter* strains comprise up to 7% of the TTX-producing bacteria, with some of the remaining genera only represented by a single strain each [20]. A link between the presence of such bacteria and dinoflagellate blooms of *Prorocentrum minimum* has been suggested when TTX was discovered in shellfish harvested in the Greek coastal waters [8], and further documented by studies on the specific mussel samples and on reference strains of *P. minimum* from the National Center for Marine Algae and Microbiota, Bigelow (ME, USA), namely the strains ccmp1529 from Equador and ccmp2956 from Johor Strait, between Singapore and Malaysia [21]. In the latter study, it was also shown that these *P. minimum* strains produced two new compounds, with m/z 265 and m/z 308, bearing a similar ion pattern and C9-base to TTX analogues and with the same effect on INa [21]. However, such a link between the presence of *P. minimum* and tetrodotoxins could not be established in England for the years 2013–2016, despite the fact that *P. minimum* (*P. cordatum*) was quite commonly detected in some areas along the southern coast of England, though with variable cell densities [22].

The link of TTX presence to toxin-producing marine bacteria has been very recently strengthened by the first discovery in the UK of two individuals of the marine nemertean *Cephalothrix simula*. This species originates from the Pacific Ocean and has been long associated with high TTX levels. Taxonomic assignment of the *C. simula* microbiome by 16S gene sequencing confirmed the prevalence of a large number of previously associated with TTX production bacterial genera, including *Alteromonas*, *Vibrio* and *Pseudomonas*. Moreover, the presence of multiple analogues of TTX, dominated by the parent TTX, was revealed by LC-MS/MS analysis of the nemertean tissue, with the total toxin concentration reaching 54 μg TTX/g tissue. In addition, the extracts of both *Pseudomonas luteola* isolated from the highly toxic *C. simula*, and *Vibrio alginolyticus* from a non-toxic native nemertean *Tubulanus annulatus*, cultured at low temperature (22 °C) were found by LC-MS/MS to contain the parent TTX, with levels quantified at 93 ng and 88 ng TTX per litre of culture, respectively [23].

A few studies have suggested that the origin of TTX is endogenous, at least in the case of certain pufferfish species, where it has been suggested that TTX possesses a defense mechanism role, and that extant bacteria are no longer involved in their biosynthesis, despite the fact that the biosynthetic genes may have been originally bacterial. Interestingly, TTX appears to be essentially absent from fauna exclusively living in freshwater habitats, and not just returning to aqueous systems for reasons of breeding [24]. On the other hand, initial studies on the micro-distribution of TTX in the New Zealand clam *Paphies australis* revealed that siphons contained the highest concentrations of TTX, while immunohistochemistry analyses showed TTX in the outer cells of the siphons, and additionally in the digestive system, foot, and gill tissue. Accumulation of TTX in the organs involved in feeding provided indicative evidence to support the hypothesis of an exogenous source, at least in the case of *P. australis* [25]. This hypothesis was further documented by depuration studies on the same species maintained in captivity on a toxin-free diet every three to 15 days for 150 days, where the siphons retained the highest amount of TTX across the entire depuration study. In the case of the digestive glands, however, the low TTX concentrations present at the start of the experiment depurated rapidly and only traces remained after 21 days [26].

The exact biosynthetic pathway and biosynthetic genes for TTX have not been elucidated to date. Due to the fact, however, that most TTX analogues are only found in either terrestrial or marine organisms, with the exception of a few analogues that are detected in both, it has been suggested that TTX biosynthetic pathways are different between terrestrial and marine animals [27]. This hypothesis is further strengthened by the recent work of Ueyama et al. [28], reporting the discovery of seven novel spiro bicyclic guanidino compounds (compounds 2–8) isolated from the pufferfish *Tetraodon biocellatus*, with six of them (2–5 and 7–8) sharing the same carbon skeleton and relative configuration as TTX. It was thus assumed that these seven compounds may be biosynthetic intermediates of TTX in marine environments and specifically precursors of 5,6,11-trideoxyTTX, since none of these compounds have been detected in toxic newts. This assumption was further supported by LC-MS analyses, confirming the wide distribution of two of these compounds (2-3) in a number of different TTX-containing marine animals, specifically five species of pufferfish (*T. biocellatus*, *Takifugu flavipterus*, *Takifugu chrysops*, *Arothron manilensis*, *Chelonodon patoca*) and one species of each for crab (*Atergatis floridus*), octopus (*Hapalochlaena lunulata*) and flatworm (*Planocerid* sp.). It was further suggested that marine TTX, its analogues and related compounds, which include compounds 2–8, are all produced by marine microorganisms and then accumulate in TTX-containing marine animals, but further screening for the presence of these TTX-related compounds in marine microorganisms would be required to confirm the proposed biosynthetic pathway to TTX, combined with an attempt to find the precursors of compound 7 and the genes for TTX [28].

## 3. Methods of Detection and Quantification

The mouse bioassay was the initial detection method for TTX, being based on its acute effects after intraperitoneal (i.p.) injection. However, ethical issues about the use of animals, together with the issues of selectivity and capability to detect and quantify analogues, have given floor to the development of more advanced, ethically acceptable and/or more targeted methods, such as in vitro bioassays, immunochemical methods and chemical-analytical methods. The most important features of the recently developed methods are summarized in Table 1, at the end of the current section.

### 3.1. Bioassays

#### 3.1.1. Cell-Based Assays

Sensitivity of the neuroblastoma Neuro-2a (N2a) bioassay to the presence of TTXs has long been documented, with the test being able to detect 0.1 ng TTX/well, and considered sufficient when applied in practice [40,41]. The N2a assay had been used in the past to screen for potential TTX-production by bacteria and sediments, but no reports on the testing of fish or shellfish were available up to the time of the EFSA opinion publication [16]. In this context, Gerssen et al. [29] used the N2a assay to verify their initial positive LC-MS/MS findings, and at the same time to test the applicability of the N2a assay as a potential alternative method for the mouse bioassay. Selection of samples was based on the LC-MS/MS results, and involved testing of two contaminated oyster samples, with TTX contents of 253 and 113 µg TTX/kg, one contaminated mussel sample quantified at 171 µg TTX/kg, and three blank shellfish samples, with the assay developed as such that a concentration of 20 µg TTX/kg shellfish could be detected. Exposure of the cells to the prepared extracts showed that all three contaminated samples were classified as suspect by the bioassay, i.e., showing an increased MTT (3-[4,5-dimethylthiazol-2-yl]-2,5 diphenyl tetrazolium bromide) activity, as indicated by absorbance measurement of the formed purple colored formazan. On the other hand, the blank shellfish samples, the chemical blank and the dimethyl sulfoxide (DMSO) (0 nm TTX) controls all showed similar low MTT activities, while a clear increase of the MTT activity was observed in the case of the positive control (100 nM TTX). Results thus indicated that the N2a bioassay could be applied for qualitative screening of large numbers of shellfish samples in order to detect the potential presence of voltage gated sodium blocker toxins, such as TTXs and Paralytic Shellfish Poisoning (PSP) toxins in shellfish. One of the advantages of this assay is the possibility to assess the toxicity of TTX analogues [42]; the main disadvantage, however, is that co-occurrence of PSP toxins (STXs, GTXs) in the same sample, even in relatively low levels, is expected to introduce false positive results, as the bioassay’s sensitivity is for STX and TTX comparable, whereas the regulated level of STXs is much higher than that proposed for TTXs [29].

#### 3.1.2. Immunoassays/Surface Plasmon Resonance Techniques

Antibody-based methods, such as competitive inhibition enzymatic immunoassay (ELISA) or surface plasmon resonance biosensor (SPR), are potentially useful for qualitative identification, but not ideal for routine screening at present, due to fact that there is no cross reactivity for all the known TTX analogues. Recent developments in TTXs detection in this type of methods comprise a nanoarray planar waveguide biosensor, initially tested on pufferfish samples [32] and the modification of a self-assembled monolayer-based Immunoassay (mELISA) previously developed for TTX detection in pufferfish [43], which has now been adapted to the analysis of oyster and mussel samples [30]. As regards the biosensor method, TTX detection was possible at levels of 0.4–3.29 μg/g puffer fish tissue in 10 min; repeatability and reproducibility were assessed at 0.4 and 0.8 μg/g, showing relative standard deviation (RSD) values below 15% and toxin recoveries within 85–115%. Cross-reactivity with some of the known TTX analogues was indicated by the quantitative results, when compared to the mouse bioassay results and the concentrations obtained by LC-MS/MS but no cross-reactivity factors were established [32]. Concerning the mELISA technique, the modifications introduced significantly improved the effective detection limits (eLOD) of the assay, with the lowest values obtained being 20 and 50 μg TTX/kg for oyster extracts without and with SPE cleanup, respectively, and about 30 μg TTX/kg for mussel extracts with both protocols, substantially below the eLOD obtained by the previous mELISA for puffer fish (230 μg TTX/kg). The mELISA showed high selectivity for TTX as no cross-reactivity of the antibody with co-occurring PSP toxins was observed. Analysis of naturally-contaminated samples, in comparison with LC-MS/MS quantifications, indicated that the mELISA method could be a promising tool for the rapid (< 2 h on the same day) detection of TTX in oyster and mussel samples, with potential application in routine monitoring programs [30].

Additionally, an electrochemical magnetic bead-based immunosensing tool has been very recently developed and tested for the presence of TTX in pufferfish tissue [31]. Evaluation of the method quality parameters indicated an LOD (established at the 20% inhibition coefficient, IC_20_), of 1.2 ng/mL and a working range (IC_20_–IC_80_) of 1.2–52.7 ng/mL. Repeatability (intra-day precision) according to Horwitz equation was acceptable, with the RSD values being 15.4 and 6.9% at 25 and 6.3 ng/mL, respectively, while this was the case also with reproducibility (inter-day precision), with the RSD values being 16.0 and 8.2% at the same TTX concentrations. It should be noted, however, that the electrochemical immunosensing tool provided higher TTX concentrations than Liquid Chromatography coupled to High Resolution Mass Spectrometry (LC-HRMS) analysis, which was attributed to the different detection principles of the techniques. LC-HRMS determines individual TTX and TTX analogues contents the analysis of which is targeted; the immunoapproach on the other hand shows the response of all the TTX and TTX analogues able to cross-react with the TTX antibody. Cross-reactivity can vary between the different TTX analogues and it is not necessarily toxicity-related [43], which means that their individual concentration and cross-reactivity with the TTX antibody, could influence to a greater or lesser extent to the TTX equivalent contents measured by the immunosensing tool. Nevertheless, it was concluded that the electrochemical immunoapproach could be useful as a screening tool to avoid false negative results, whereas in case of a positive result, a complementary analysis of the sample would be required for confirmation purposes [31].

### 3.2. Spectrofluorimetric Methods

A sensitive detection method for TTX based on up-conversion fluorescence using a microfluidic aptasensor was very recently developed by Jin et al. [33], with a detection limit of 0.06 ng/mL. Magnetic Fe_3_O_4_ nanoparticles were modified with a TTX aptamer to form a Fe_3_O_4_/aptamer complex. The self-assembling of carbon dots (CDs) and the aptamer generated Fe_3_O_4_/aptamer/CDs nanocomposites, which exhibited down-conversion fluorescence and up-conversion fluorescence (UCF) emissions simultaneously. When excited at a wavelength of 780 nm, the UCF (peaked at 475 nm) of nanocomposites increased regularly with the increase of TTX concentration, with the UCF peak intensities increasing almost linearly with the increase of Log TTX concentration within the range from 0.1 ng/mL to 0.1 mg/mL and with a high linearity coefficient (R^2^) of 0.9975. A novel UCF turn-on probe of TTX was developed using the nanocomposites and experimental evaluation of potential interferences confirmed the highly selective and sensitive UCF responses. The method was initially tested in TTX-spiked real biological fluids of human body (gastric juice, human serum and urine from healthy volunteers) and results revealed high detection recoveries ranging between 96%–104% with RSDs of 1.43%–4.21%, over a spiking range from 1 ng TTX/mL to 100 μg TTX/mL, thus indicating that the novel TTX-probe could be a promising alternative for TTX detection in real biological sample analysis [33].

### 3.3. Chemical-Analytical Methods

Recommendations included in the EFSA opinion [16] highlighted the importance of obtaining more data on TTX occurrence using EU approved and validated chemical-analytical methods. In this context, the need for certified standards and reference materials for TTX and its analogues was also pointed out. A certified standard solution for TTX (25.9 ± 1.3 μg/g) and 4,9-anhydroTTX (2.99 ± 0.16 μg/g) in 1 mM acetic acid, also containing small (non-certified but quantified) amounts of 4-epiTTX and 11-deoxyTTX and minor quantities of norTTX and trideoxyTTX, has been available since 2012 (Laboratorio Cifga, Lugo, Spain) and has been used increasingly for the establishment of validated methods. Very recently, the National Research Council of Canada (NRC, Halifax, Canada) announced the production of a TTX standard solution at 6.8 μg/g in 1 mM acetic acid and of a pilot scale multi-class mussel tissue matrix reference material for polar marine toxins including TTX, PSP toxins and domoic acid (DA), which are expected to be commercially available in the near future [44].

The progress made in the past two years towards establishment of validated methods for future implementation in the monitoring programmes of EU member states has been significant, while also the first proficiency test at EU level using naturally TTX-contaminated materials has been organized between July and September 2018 by the EU reference laboratory for Marine Biotoxins (EURLMB, Vigo, Spain). Most of these are single-laboratory validated analytical methods, employing the LC-MS/MS technology, and can either simultaneously detect TTXs and STXs or have a multitoxin approach, while a number of High Resolution LC-MS/MS (LC-HRMS/MS) methods and a validated multiclass capillary electrophoresis–tandem mass spectrometry (CE-MS/MS) method have also been developed.

#### 3.3.1. Single Analyte Group LC-MS/MS Methods

A single-laboratory validation was conducted for the Hydrophilic Interaction LC coupled to tandem MS (HILIC-MS/MS) analysis of TTX in common mussels (*Mytilus edulis*) and Pacific oysters (*Crassostrea gigas*), two of the bivalve species found to contain TTXs in the United Kingdom in recent years [34]. The method involves a single-step dispersive extraction in 1% acetic acid, followed by a carbon SPE cleanup step before dilution and instrumental analysis and has been developed as a rapid tool for the quantitation of the parent TTX and the analogs 4-epiTTX; 5,6,11-trideoxyTTX; 11-norTTX-6-ol; 5-deoxyTTX; and 4,9-anhydroTTX; however the combined acquisition of PSP toxins is also possible, as indicated by Boundy et al. [45]. All method performance characteristics assessed for analysis of the parent TTX (specificity, linearity recovery, ruggedness, repeatability, matrix variability, and within-laboratory reproducibility) were found within the acceptable levels. Correlation coefficients for linearity were >0.999 over the full six-point calibration range for each toxin in the solvent, mussel, and Pacific oyster matrixes. Mean TTX recoveries for the three concentration levels studied (20, 100 and 200 μg/kg shellfish tissue) ranged from 70% to 77% for mussels and from 76 to 84% for Pacific oysters, with a higher mean recovery recorded for oysters at the high spiking concentration in comparison to mussels. The LOD, LOQ and LOR values were comparable for mussels and oysters and were considered fit-for-purpose taking into account the EFSA proposed limit of 44 μg/kg. More specifically, the LOD was approximately 0.25 μg/kg shellfish tissue for both matrices, LOQs were found to be 0.79 and 0.76 μg/kg for mussels and Pacific oysters, respectively, and the LORs were set to 2 μg/kg for both matrices. Additionally, aspects of method performance (LOD, LOQ, and within-laboratory reproducibility) were found to be satisfactory for three other TTX analogs (11-norTTX-6-ol, 5-deoxyTTX, and 4,9-anhydroTTX). The method expanded uncertainty (k = 2) for TTX was calculated at 36% and 51%, for mussels and oysters, respectively, while preliminary assessment of the method uncertainty for the above three analogues indicated comparable or lower values than those of the parent TTX. Cumulatively, the method was found to be practical and suitable for use in regulatory testing, providing rapid turnaround of sample analysis [34].

A similar, slightly modified protocol was intralaboratory validated by the EURLMB in 2017, and has been used as a Standard Operating Procedure (SOP) for the determination of TTXs at European level [35]. The protocol also involves a graphitized carbon SPE clean-up and requires an 11-min run using HILIC-MS/MS. Quantification was performed using matrix matched calibration curves with linearity being generally above 0.99. Recoveries obtained for mussels were assessed at seven spiking levels (10 μg/kg, 20 μg/kg, 44 μg/kg, 100 μg/kg, 900 μg/kg, 4 mg/kg and 10 mg/kg) and were within the range of 91.6 to 109.5%. With regard to oysters, four spiking levels were studied (10 μg/kg, 25 μg/kg, 44 μg/kg, 100 μg/kg), with the recoveries obtained being much lower and ranging between 69.3% and 79.6% [35].

An Ultra Performance LC-MS/MS (UPLC-MS/MS) method for the determination of TTXs in shellfish was implemented by Gerssen et al. [29], also based on the method of Boundy et al. [45]. Apart from the parent TTX, the following TTX analogues were included in the method: 4-epiTTX; 5,6,11-trideoxyTTX; 11-norTTX-6-ol; 4,9-anhydroTTX; 5- and 11-deoxyTTX. Certified TTX standards were used to prepare matrix-matched calibration curves, with the linearity acceptability criterion set at above 0.990. The LOD and LOQ values were determined in fortified shellfish extracts and found to be 3 μg/kg and 20 μg/kg, respectively [29].

#### 3.3.2. Multi-Analyte Toxin Group LC-MS/MS Methods

The majority of the HILIC-MS/MS methods developed for the determination of TTXs are able, in principle, to simultaneously detect PSP toxins by adjusting the LC conditions and MRM transitions monitored. However, methods applying a more “multi-analyte approach” have increasingly gained interest, due to the possibility to save time and costs. In this context, an LC-MS/MS method for the detection and quantification of all EU-regulated marine toxins (both lipophilic and hydrophilic) in a single 19-min run, with the same LC and MS conditions has been recently developed. The method allowed identification and quantification of the hydrophilic toxins PSPs, DA and TTX and analogues, as well as of the regulated lipophilic toxins (OA group, YTXs, AZAs), with significantly improved LODs and LOQs in comparison to other official and validated methods. Repeatability was within the acceptable HorRat values, while RSDs were generally low for most of the toxins included. The method was specific and linear over the full calibration range and accurately determined sample toxicity and toxins concentrations for all the toxins included. As regards TTXs, the LOQs obtained were slightly higher than those of other validated methods, with a maximum of 1.56 μg TTX/Kg of shellfish, but were more than sufficient compared to the EFSA limit proposed [36].

#### 3.3.3. High Resolution Mass Spectrometry (HRMS) Methods

Triple quadruple MS methods are considered ideal for routine analysis of toxins, including TTXs, providing sensitive and selective quantitation. On the other hand, the introduction of Fourier transform mass spectrometer (FTMS) Orbitrap instruments has resulted in an increasing interest towards the application of HRMS methods to several food toxins, drugs and pesticide residues in food and related matrix in the last decade. HRMS methods have been recently used in TTX analysis, mostly for identification purposes [29,46]; however, application of a validated HRMS method for TTX identification and quantification in puffer fish and shellfish, providing accurate results, even with no use of standards for TTX analogues, has been described by Bane et al. [37]. The study plan was to investigate the presence of both known and unknown TTX analogues, using characteristic fragment ions of the TTX family. All the intense precursor ions were scanned during the full scan MS experiment, whereas in the data dependent (dd) MS2 scan mode, the precursor ion mass list was set to analyze TTX analogues usually occurring in puffer fish and shellfish including: m/z 272.12 (5,6,11-trideoxyTTX and 4-epi-5,6,11-trideoxyTTX), m/z 288.12 (5,11-dideoxyTTX and 6,11-dideoxyTTX), m/z 290.10 (11-norTTX-6-(S)-ol and 11-norTTX-6-(R)-ol), m/z 302.10 (4,9-anhydroTTX and anhydroTTX), m/z 304.11 (5-deoxyTTX and 11-deoxyTTX), m/z 320.11 (TTX and4-epiTTX), m/z 336.27 (11-oxoTTX) and m/z 423.42 (4-S-CysTTX). Unknown analogues were detectable only if their precursor ions intensities were higher than those of the sample matrix ions. The method was validated according to the criteria of the Commission Decision 2002/657/EC (Decision 2002/657/EC). Method specificity was indicated by the general stability of retention times for both the parent TTX (in standard) and its analogues (in samples), at least for 4-epiTTX, 4,9-anhydroTTX, 11-deoxyTTX, 5-deoxyTTX 5,6,11-trideoxyTTX, 11-norTTX-6(R)-ol, 11-norTTX-6(S)-ol, 5,11-dideoxyTTX and 6,11-dideoxyTTX. LOD and LOQ were calculated at 0.008 μg/mL (0.041 μg/g) and 0.027 μg/mL (0.136 μg/g), at S/N 3 and 10 respectively, by spiking TTX standard into TTX free mackerel fish extracts. As regards trueness, the relative errors for controls, also prepared by spiking TTX standard into TTX free mackerel fish extracts, were 8.90% (2.5 μg/mL) and 20.72% (0.25 μg/mL), indicating an acceptable accuracy of the method, while intraday RSD (*n* = 3; 2.78%–7.92%) and interday RSD (*n* = 9; 8.13%–14.23%) showed the good repeatability and reproducibility of the method [37].

Additionally, a multi-toxin LC-HRMS analysis method was developed to confirm and quantify TTXs in the pufferfish *L. sceleratus* [38], including both analogues already described for the species but also other possible analogues described in the literature [1]. In general, quantifications by LC-HRMS (LOD = 0.05 mg/kg and LOQ = 0.1 mg/kg) agreed with those obtained by LC-MS/MS in the same work, but most importantly LC-HRMS analysis allowed the identification and quantification of some analogues (4,9-anhydro-5,6,11-trideoxyTTX and 4,4a-anhydro-5,6,11-trideoxyTTX) present in high concentrations, which were not included in the LC-MS/MS analysis method [38].

#### 3.3.4. Capillary Electrophoresis–Tandem Mass Spectrometry (CE-MS/MS)

A CE–MS/MS method has been recently developed for the multiclass analysis of polar marine toxins, i.e., paralytic shellfish toxins, tetrodotoxins and domoic acid in seafood, using a custom-built interface and a novel, highly acidic background electrolyte (5 M formic acid). The method was applied to a wide range of regulated and less common toxin analogues, exhibiting high selectivity between toxin isomers and matrix interference. The LODs obtained in mussel tissue were 0.0052 mg/kg for TTXs, 0.160 mg/kg for domoic acid, and between 0.0018 and 0.120 mg/kg for paralytic shellfish toxins, and good linearity was observed in all cases. Analysis of shellfish matrix reference materials and spiked samples demonstrated good accuracy and precision for all the toxins analyzed. Regarding specifically TTX detection, quality parameters were assessed using a prerelease certified standard solution (CRM-TTX) and sea slug (*Pleurobranchaea maculata*) samples naturally containing TTX and several analogues and in general good agreement was obtained when comparing values of the spiked extracts with those found by the CE–MS/MS analysis [39].

## 4. Recent Occurrence Data in European Waters

After adoption of the EFSA opinion on TTXs, several new occurrences of TTXs in aquatic organisms have been reported from different countries relevant to European waters. The main findings are summarized in Table 2 and described in detail in this section, with a special emphasis on marine bivalves and gastropods.

### 4.1. Italy

The presence of TTXs in Italy has been documented both in the northern and southern coastal areas. In May 2017, six shellfish samples (mussels *Mytilus galloprovincialis* and wedge clams *Donax trunculus*) collected in the Northern Adriatic Sea (Veneto and Friuli Venezia Giulia) were analyzed by hydrophilic interaction liquid chromatography coupled to mass spectrometry (HILIC-MS/MS) for both PSP toxins and TTXs, after an initial screening by the AOAC 959.08 mouse bioassay (MBA) resulting in survival times as low as 8 min [47]. In one of the mussel samples collected in Ficariol San Piero (Lagoon of Marano), TTX was detected at a concentration of 541 μg/kg, whereas no further contamination was recorded in mussels samples harvested one week later at the same site and no PSPs were detected. The presence of TTX in that sample was also confirmed using HILIC in combination with high resolution multiple stage mass spectrometry (HRMS^n^, *n* = 3), resulting in a TTX concentration of 413.2 μg/kg. The shellfish harvesting area of Ficariol San Piero is a natural bank located inside the lagoon of Marano, a semi-enclosed tidal basin with few marshes and islands above sea level. The lagoon has a maximum depth of 1–2 m, with the exception of the main input channels, which reach depths of up to 6 m, whereas at the time of sampling (22/05/2017) the water temperature was 19.7 °C and salinity was 33.4 psu. Interestingly, TTX contamination was also detected in two mussel samples, which were positive in the AOAC 959.08 MBA and harvested in shallow estuarine waters (1–2 m depth), from the exact same area of Ficariol San Piero and at the same time period (22/05/2018 and 04/06/2018). No PSPs were detected in both samples by HILIC-MS/MS; on the contrary TTXs were found at levels of 216 μg/kg and 86 μg/kg, respectively, with the water temperature ranging between 18–20 °C [48].

Where presence in the southern Italian coasts is concerned, Dell’ Aversano et al. [46] have conducted a survey during the spring/summer periods (from April to July) of 2015–2017 in mussels (*M. galloprovincialis*) and clams (*Venerupis decussata*) collected from sampling stations located in Suracuse bay (Ionian Sea, Mediterranean Sea) on the occurrence of both PSPs and TTXs, with the latter being analyzed by two different methods, Ultra High Performance (UHP) HILIC-MS/MS and High Resolution (HR) HILIC-MS/MS. In the UHP HILIC-MS/MS method, apart from the parent toxin which was quantified against a certified reference standard (Laboratorio CIFGA, Lugo, Spain), a number of TTX analogues were also incorporated, including 4-epiTTX, 5,6,11-trideoxyTTX; 11-norTTX-6-ol; 4,9-anhydroTTX and 5-deoxyTTX/11-deoxyTTX. The analogues were semi-quantified using the TTX calibration and assuming an equimolar response, whereas results were not adjusted for recovery as all quantifications were performed using matrix-matched standards. The presence of TTXs was confirmed by UHP HILIC-MS/MS in 14 out of the 25 analyzed samples, with the parent TTX being the only toxin detected, except for the sample with the highest concentration, which also contained 4-epi TTX. The levels reported were generally low, with a maximum concentration of 6.4 μg/kg, while 10 out of the 14 positive samples, which were all mussels, contained TTX above the method’s Limit of Reporting (LOR) of 2 μg/kg and the remaining four at levels above the method’s calculated Limit of Quantification (LOQ) of 0.8 μg/kg. However, the reported TTX contamination was not possible to confirm by HR HILIC-MS/MS. Although no statistical analysis was conducted in that work, certain trends are noteworthy, such as: (a) in most cases TTXs co-occurred with very high levels of PSPs in the same sample, with the highest TTX concentration of 6.4 μg/kg found in the sample with the highest total PSP level, exceeding 10,000 μg STX-diHCl eq/kg by all three analytical methods, and (b) most of the TTX contaminated samples, and actually those with the highest levels, were harvested from the harbor area, where they were found attached at ca. 1 m depth [46].

### 4.2. Netherlands

After the initial surveys conducted when the issue of TTXs presence in European shellfish first emerged in 2015, the Netherlands established a sanitary survey, being a regular weekly sampling for evaluating TTXs presence by an LC-MS/MS method, in terms of both official control and research, at a seasonal basis from June to October in 14 different production sites. Regulatory measures were imposed based on the precautionary principle when the TTX levels found exceeded the decision limit, which was: (a) the method LOQ of 20 μg TTX/kg shellfish in 2016 and (b) the EFSA proposed limit of 44 μg TTX/kg shellfish starting from 2017 [29]. The Dutch sanitary survey has so far involved the sampling and analysis of four shellfish species: mussels (*M. edulis*), oysters (*C. gigas*, now *Magallana gigas*), razor clams (*Ensis* spp.) and cockles (*Cerastoderma edule*), with a significant number of samples analyzed yearly (Table 3). It should be noted that some of the data on TTXs presence in Dutch shellfish harvested in 2015 and 2016 have already been taken into consideration in the TTX EFSA opinion. However, they have also been included in Table 1, due to the fact that certain differences in the reporting criteria have been later introduced (e.g., different method LOQ), as well as for providing a more complete assessment of the trends observed through the years.

According to the results available from 2015 until October 2018, certain patterns regarding TTX accumulation, depuration and distribution are evident. Only two sampling areas, located in the Eastern Scheldt were affected, i.e., east and north, with the highest TTX contents (clearly observed over the years in oysters (max. 253 μg TTX/kg in 2016) collected from the Eastern Scheldt east, followed by rope cultured mussels (max. 101 μg TTX/kg in 2016) harvested from the Eastern Scheldt north, while so far no TTX has been detected in razor clams and cockles. It should be noted, however, that although from all the data collected it seemed that oysters were more susceptible than mussels for TTX accumulation, the two species were not harvested from adjacent plots. In this context, additional mussel samples were taken from the Eastern Scheldt east, as close as possible to the oyster production plots. Analyses confirmed that oysters indeed contained higher concentrations of TTX than mussels (e.g., 101 and 218 μg TTX/kg, in mussels and oysters, respectively, collected at 29-30/06/2016), with this difference steadily observed over the course of 2015 to 2017 [29,49]. The vertical distribution of TTX contents was also assessed in rope cultured mussels, as the samples taken for the official control program were pooled samples with equal numbers of shellfish from top, middle and bottom of the rope. In several occasions top, middle and bottom samples were analyzed individually to determine if there are variations between sampling heights. Results indicated that no significant differences were observed between samples taken from the three different positions [29].

Seasonal occurrence of the toxic episodes over the years has been quite consistent, with TTX positive samples generally present in the summer months (June–August), an increase in TTX concentrations being observed during late June, followed by a rapid decline in July, as evidenced by intensification of the sampling scheme after the first positive findings. The most intense episode was that of 2016, where the highest concentration of 253 μg TTX/kg was reported, followed by 2015, while the levels detected in 2017 and 2018 were much lower. With regard to the presence of TTX analogues or other toxins, analyses conducted by HR-MS/MS where 31 TTX analogues and approximately 800 other marine and freshwater toxins were included, indicated that co-occurrence of other toxins was negligible and confirmed that the parent TTX was almost exclusively the only TTX present, with the exception of one sample containing 4-epiTTX, estimated by LC-MS/MS at the level of 14.5 μg TTX-eq/kg using the TTX calibration curve, with TTX being present at 96 μg/kg in the same sample [29].

### 4.3. Spain

Studies aiming at the evaluation of TTXs occurrence in bivalves, according to the EFSA recommendations, and at the same time investigating its association with the presence of high *Vibrio parahaemolyticus* concentrations were undertaken in 2017 and 2018 in the Spanish coastal areas [50,51]. Sampling was conducted in the three main Galician Rias (Vigo, Pontevedra and Arosa), located in the Atlantic west coast of Spain, in which the production of bivalves, and in particular mussels, is the most representative in the EU, also being among the main areas of mussel production in the world and involved six different types of bivalves (mussels, oysters, cockles, clams, scallops and razor clams). Mussels in that area are grown on 15 m long ropes hung from floating platforms, while the infaunal bivalves (oysters, scallops, razor clams, cockles, and clams) are harvested in the estuarine zones around the Rias. In 2017, a total number of 1279 bivalve samples collected between January and September, were initially subjected to microbiological analysis for the detection and isolation of *Vibrio* spp. Results confirmed the presence of *Vibrio* spp. in 286 of the samples, with the largest proportion involving pelagic samples (mussels) compared to the infaunal samples (258 vs. 28 samples, respectively). Samples positive to the presence of *Vibrio* spp. were further screened for TTX toxicity by a neuroblastoma cell-based assay (N2a), while TTX in the same samples was also determined by HILIC-MS/MS. TTX toxicity by N2a was found in only 2 out of the 286 samples: cockles from Ria of Pontevedra collected in June 2017 and oysters from Ria of Arosa collected in February 2017. Analysis by HILIC-MS/MS showed a good agreement with N2a, as TTX was detected only in the above two infaunal cockle and oyster samples. Only the parent TTX was detected at levels of 2.3 μg/kg in cockles and below the method LOQ (0.9 μg/kg) in the oyster sample, with the quantification based on a matrix matched calibration curve, while no presence of TTX or any of its analogues was found in any of the mussel samples. Water temperatures during sampling of the above cockles (June) and oysters (February) were at 17 °C and 13.5 °C, while salinity in both cases was 35 psu [50]. In the second phase of the study, conducted during May to July 2018, all of the 72 bivalve samples analyzed on the same basis were found negative to the presence of TTXs [51].

### 4.4. France

Investigation on the presence of TTXs in French bivalve molluscs was conducted during 2018 to estimate occurrence and contribute towards a more reliable exposure assessment using an LC-MS/MS method based on the harmonized protocol of the European Reference Laboratory for Marine Biotoxins for the determination of tetrodotoxins [35], with a LOD of 8.0 μg TTX/kg and a LOQ of 15.0 μg TTX/kg. Sampling involved the eleven geographical points of the French monitoring system for emerging toxins in order to cover all the French metropolitan coasts (from the English Channel, passing through Atlantic Ocean and the Mediterranean Sea). A total of 132 samples were collected and analyzed throughout the year 2018, on the basis of one sample per point and per month, with eight points sampled for mussels, two points for oysters and one for both molluscs. Results indicated the absence of the parent TTX and six of its analogues included in the method (4-epiTTX; 11-deoxyTTX; 5-deoxyTTX; 4,9-anhydroTTX; 6,11-dideoxyTTX; and 5,6,11-trideoxyTTX) at levels above the LOD in all samples analyzed [52].

### 4.5. Ireland

Publication of the EFSA opinion triggered investigations on the occurrence of TTXs also in Ireland. Studies were initiated in 2017 by the Marine Institute, which started by analyzing archive samples from 2015, by an LC-MS/MS method. As the presence of TTX is considered more likely in the summer months, the first two samples submitted from every production area for the months of June, July, August and Sept 2015 were selected, reaching a total of 316 samples from all shellfish species harvested in the relevant areas, including all gastropods and grazers. A number of samples from 2012–2014 from Castletownbere were also tested and, additionally, the area of Cork Harbour was chosen be intensively analyzed as a full year test site due to its shallow waters (having been associated to the presence of TTX), southern location and the availability of both oysters and mussels. By the end of 2017, a total of 356 archive samples from the years 2012-2015 had been analyzed, of which none exceeded the method LOQ. As a result, analysis of archive samples from 2016, including one sample per month from June–September from Cork Harbour was decided to further continue the investigation [53,54] and until June 2018 a total of 508 samples had been tested with similar results, i.e., no presence of TTX above the method LOQ [55,56].

### 4.6. United Kingdom (UK)

After the initial data on TTXs presence in bivalve molluscs (mussels and Pacific oysters) harvested during the years 2013–2014 from two sites located in south England, UK [7], which had been taken into account in the TTX EFSA opinion, further studies were undertaken to expand the testing to other geographical regions of the UK, in order to investigate the prevalence and both geographical and temporal variability throughout the country. As a full screen for the presence of TTXs of all shellfish samples submitted for official control (OC) testing was impossible, taking into account the high sample numbers involved (>3500 per year) and limitations on spare resources, including suitable laboratory instrumentation, a multi-stage approach was adopted. At first, analyses were conducted by LC-MS/MS on selected OC samples taken from around the entire UK coast, including England, Scotland and Wales, between March and October 2014, to provide preliminary indications of TTXs prevalence and associated spatial and temporal variability. Secondly, all OC shellfish samples collected in July 2015 were analyzed, as the prevalence of TTXs seemed higher the in summer. Thirdly, in order to allow for continuous long-term assessment in the most affected areas, a number of samples originating from selected sites in the south of England were tested between May 2015 and November 2016. Finally, samples harvested in Northern Ireland were analyzed during 2016 [22].

#### 4.6.1. England

Samples from the English coasts, collected across the whole of England during the warmer months of July to October 2014 and only from the south part during May to October 2015, consisted of mussels (*M. edulis*), Pacific oysters (*C. gigas*), native oysters (*Ostrea edulis*), cockles (*C. edule*), hard clams (*Mercenaria mercenaria*), surf clams (*Spisula solida*) and king scallops (*Pecten maximus*), both whole and preprocessed (adductor and roe only). Moreover, the monitoring was continued after October 2015 in six selected areas in the southern England, which showed the highest prevalence for TTX-positive shellfish during 2014–2015, in order to obtain a long-term assessment in a 1.5 year span (May 2015–November 2016). TTXs were detected in 55 (12%) out of the 477 shellfish samples collected in classified English production areas between 2014 and 2016 and tested in this study, at total TTXs concentrations exceeding the limit of reporting (LOR) of 2 μg/kg. Fourteen samples (3.0% of total samples) contained total TTXs above 20 μg/kg (Dutch limit in 2016), although two additional samples contained total TTXs very near to this limit (>19.5 μg/kg). All of these samples were harvested from shellfish sites located in the south coast of England. The highest total TTXs content reached 253 μg/kg in a Pacific oyster sample harvested in July 2015. TTXs were detected in both winter and summer, however all total summed concentrations above 20 μg/kg were found in June, July and August, although one of the mussel samples with a total TTXs value of 19.9 μg/kg was collected in January 2016. As regards seasonality of TTX occurrence in shellfish, despite the fact that differences in total TTX contents and longevity of toxicity were evident between the six main southern regions which were regularly tested between May 2015 and November 2016, there were strong indications of increased toxicity during the summer months in all six areas. Solid indications concerning uptake or depuration rates in the majority of shellfish harvesting areas found to contain TTX positive samples, were not easy to obtain, as the sampling frequencies were not adequately high. It is noted, however, that the tentative patterns observed were not consistent; in one site where hard clams were tested TTXs concentrations increased from the LOR to a maximum of 72 μg/kg over a three-week period and decrease to approximately 10 μg/kg required further three weeks. On the other hand, a rapid drop in TTXs concentrations was observed in another site over a two-week period in mussels, followed by a 40% increase in the subsequent two weeks, before concentrations dropped below LOR, indicating a possible species and/or local environmental factors effect [22].

It is noteworthy, however, that in England TTXs were detected in various species including mussels, Pacific oysters, native oysters and hard clams, but not in any sample of cockles, surf clams or in any of the non-OC king scallop samples, collected according to the wild Pectinidae verification process. During 2014–2015, only mussels, Pacific oysters and hard clams had total TTXs concentrations above 20 μg/kg, while in 2016, samples containing TTXs > 20 μg/kg were found in all four species, with the highest concentrations quantified once more in Pacific oysters. A thorough evaluation of uptake differences between different species was not possible, however, as collection of only one representative species was possible at each OC monitoring site [22].

#### 4.6.2. Scotland

In total, 670 Scottish samples, of which 660 OC samples from routinely monitored classified production areas and 10 verification scallop samples, were collected and tested between March and October 2014, as well as during July 2015. TTXs concentrations exceeded the LOR (2 μg/kg) in only two samples, one of which (mussels collected in July 2014) contained TTXs at levels above 20 μg/kg (26 μg/kg) [22].

#### 4.6.3. Wales

None of the twenty-eight shellfish samples (cockles and mussels) collected from routinely monitored classified shellfish production areas in Wales during July to September 2014 and July 2015 contained TTXs above quantifiable levels. Trace levels of TTX were detected in only one Welsh sample of cockles, but did not exceed the LOR of 2 μg/kg [22].

#### 4.6.4. Northern Ireland

No TTXs were detected in any of the fifty-seven shellfish samples (mussels and Pacific oysters) collected between May and September 2016, from selected classified shellfish production areas of Northern Ireland and tested in the study of Turner et al. [22].

#### 4.6.5. Toxin Profiles

With regard to TTX analogues’ presence, the dominant one found across all quantifiable samples was the parent TTX toxin, representing a mean of 90% of the total TTXs concentrations quantified throughout all the positive samples in the three years of the study (2014–2016). Additional analogues detected were 4-epiTTX in samples with higher TTXs contents, 5,6,11-trideoxyTTX and 4,9-anhydroTTX, with mean proportions of 7%, 29% and 7%, respectively, whereas two samples from 2016 were found to contain low concentrations of 5-deoxyTTX. It was evident that the proportions of TTX analogues, although dominated by TTX, were variable. Some yearly differences were also noted, with the near exclusive presence of the parent TTX in positive samples collected during 2014 and 2016, but with higher relative proportions of other analogues observed in 2015, mainly of 5,6,11-trideoxyTTX. On the other hand, the total profile calculated only from samples with TTXs concentrations above 20 μg/kg showed similar results, thus indicating that the relative proportions of 5,6,11-trideoxyTTX and 4,9-anhydroTTX were not related to concentration. Moreover, no species related differences were noted in the occurrence of different TTX analogues, with 4-epiTTX, 5,6,11-trideoxyTTX and 4,9-anhydroTTX all detected in clams, oysters and mussels. Finally, all samples containing TTXs, except for one harvested in 2016, with a very low 5-deoxyTTX concentration (2.88 μg/kg), were found to contain the parent TTX itself, so there was no evidence for shellfish containing only TTX analogues, without the parent toxin being present [22].

#### 4.6.6. Environmental Effects

Water temperature recordings obtained during sampling were compared to the data on TTXs concentrations, resulting in a general indication that TTXs were typically present in shellfish collected from areas with water temperatures of ≥ 15 °C, with few exceptions. Despite the facts that no statistical correlations were established between water temperature and total TTX contents and also that no presence of TTXs was detected in a large number of shellfish samples harvested in water above 15 °C, this temperature seemed to be the threshold above which TTXs were more likely to occur in shellfish tissue. Moreover, water depth seemed to also play an important role, taking into account that that 51 out of the 55 TTX-positive samples (93%) originated from inter-tidal or shallow water environments (0–5 m depth), while the four remaining ones were all harvested from the same medium-depth location (5–20 m depth). Finally, where salinity is concerned, 45 out of the 55 TTX-positive samples (82%) were collected from estuarine shellfish beds, presumably associated with lower salinity levels in comparison to open marine waters. Of the remaining 10 samples, four were collected from the same deep-water sites as described above, whilst the other six, which were found to contain only low TTXs concentrations (< 5 μg/kg), were of riverine origin [22].

### 4.7. Turkey

Although no studies have been conducted in Turkey regarding TTXs occurrence in bivalve molluscs and gastropods, the presence of TTXs in Turkish waters has been already documented due to the successful establishment of the alien toxic pufferfish *L. sceleratus*, also known as the silverstripe blaasop [57]. According to the results of two recent studies [58,59], though, two additional alien toxic pufferfish species, the yellow spotted pufferfish (*Torquigener flavimaculosus*) and the Suez puffer *Lagocephalus suezensis* collected in Turkish waters have been shown to also contain high concentrations of TTXs. Despite the fact that at present the occurrence of TTXs in pufferfish has not been directly related to the occurrence of TTXs in shellfish, this source of increased TTXs incidence in European waters is of major concern and should be taken into account in any relevant risk assessments.

## 5. Hazard Identification and Characterisation

### 5.1. Toxicokinetics

One of the important conclusions of the EFSA opinion on TTXs with regard to toxicokinetics was the availability of limited information about absorption and excretion of TTX and its analogues in humans as well as the lack of data with regard to its distribution and metabolism [16]. A recent investigation of a puffer fish poisoning incident in New Caledonia, involving three individuals, of which one was deceased and two were hospitalized, provided further evidence on this issue [60]. Urine, serum and plasma samples from the two intoxicated patients and one post-mortem plasma sample from the deceased person were collected 17–45 h and 17 h after ingestion of the boiled pufferfish, respectively, and their multi-toxin profile (TTX and TTX analogues) was evaluated by liquid chromatography-tandem mass spectrometry (LC-MS/MS) analysis. All urine samples contained the parent TTX, as expected; however, a multi-toxin profile was revealed in all urine samples, with the presence of also 4-epiTTX, 4,9-anhydroTTX and 5,6,11-trideoxyTTX analogues. It is noteworthy that whereas 5,6,11-trideoxyTTX was the major analogue found in urine from one of the patients collected ∼17h after the puffer fish ingestion, the parent TTX was the major compound found in urine from the same patient collected ∼45h after the fish ingestion and also in the urine sample from the second patient, collected ∼42h after the fish ingestion. The concentrations of TTX and TTX analogues obtained could suggest the metabolism of 5,6,11-trideoxyTTX to TTX and its equivalent analogues 4-epiTTX and 4,9-anhydroTTX, a pathway already suggested previously by Yotsu-Yamashita et al. [27]. A decrease of TTX and all the analogue concentrations was observed with time (hours after fish consumption), but the TTX metabolic pathway could not be concluded with certainty, as differences in the metabolic profile could also be explained by the different stability of TTX analogues. A correspondence between the total TTX contents of urine samples, adjusted by the application of toxic equivalence factors (TEFs) to obtain TTX equivalent concentrations, and the clinical symptoms of the patients was also observed. As regards the serum and plasma samples of the hospitalized patients, neither TTX nor TTX analogues were detected above the LODs, indicating the rapid elimination of TTX into urine. On the other hand, both TTX and 5,6,11-trideoxyTTX were detected in the post-mortem plasma sample of the deceased person, with the major one being 5,6,11-trideoxyTTX; however due to the low toxicity of the latter compared with TTX and the very high TTX concentration found, it was evident that TTX was the main causative agent of this fatal case [60].

Tissue distribution of TTX has also been investigated in a fatal TTX poisoning case of a 70-year old man in Japan, who died in hospital about 40 h after ingestion of boiled puffer fish [61]. Serum, urine and post-mortem specimens, specifically cardiac blood, stomach contents, lung, liver and kidney, were analyzed by LC-MS/MS. While the patient was still alive, TTX concentrations in serum and urine declined fast by 60.7% and 83.6%, respectively, as indicated by two consecutive samples drawn two hours and twenty minutes apart. As regards TTX presence in post-mortem samples, the highest TTX concentrations were detected in kidneys, followed by stomach contents, liver, lung and cardiac blood. It is noteworthy, however, that in this particular case the detected TTX levels in blood and urine were lower than those reported as fatal by the literature. This was attributed to the fact that the decedent was an elderly person with evidence of cardiovascular disease (coronary artery stenosis) revealed by the autopsy. It was thus concluded that the heart condition and advanced age were contributing factors in his death by relatively moderate levels of TTX [61]; such influences should be definitely taken into account when uncertainty factors are applied within the process of risk assessment.

TTX absorption, distribution, metabolism and excretion (ADME) was also studied using radiolabeled TTX. Single intramuscular dosing of 6 μg/ (16 μCi/kg) 11-[^3^H]TTX in Sprague-Dawley rats resulted in similar pharmacokinetics of plasma total radioactivity in male and female rats, with the maximum radioactivity (5.56 ng eq/mL) reached in 10 min, while plasma [^3^H]TTX was non-detectable after 24 h. Total radioactivity showed a mean recovery rate of 69.35% between 0 and 72 h. A proportion of 51.16% of the radioactive dose was recovered in the urine, in contrast to only 0.43% excreted in bile, indicating that urine excretion is the predominant route of elimination, while 3.87% was recovered in the feces, 10.01% in the cage wash and 4.31% in the carcass. After 72 h, the radioactivity of all samples was lower than the LOQ. Average total radioactivity in the stomach, lungs, kidney and intestines was higher than that observed in plasma concentrations at the 0.5 h point. On the other hand, metabolite analysis of plasma, urine and feces samples demonstrated the presence of only oxidized TTX. As a conclusion, TTX was found to be rapidly absorbed and excreted in rats [62].

### 5.2. Toxicity

#### 5.2.1. Acute Toxicity

A Dutch in vitro study on the acute inhibitory effects of TTX in rat and human neuronal networks [63], published soon after the EFSA opinion, provided further information on the regularly used uncertainty factors. Definition of an ARfD, according to the regular procedures would require selecting the NOAEL for the most critical endpoint in an acute animal study and dividing it by an uncertainty factor of 10 for interspecies differences (between the experimental animals and human) and by another uncertainty factor of 10 for intraspecies differences (between humans), and this approach was actually the one used by EFSA. The results of Kasteel et al. [63], however, showed that TTX was roughly equipotent in both the rat and human in vitro models used, indicating that interspecies differences are limited in the case of TTX and that experimental animal (rat) data for TTX could play a more prominent role in human risk assessment of TTX. According to their conclusions, a human acute reference dose of 1.33 μg/kg body weight was derived, which corresponded to a maximum concentration of TTX in shellfish of 200 μg/kg. Subsequently, at an expert meeting on May 2, 2017, with Dutch and Belgian experts [64], it was concluded that the information of that in vitro study results in a reduction of uncertainty of 2.5, with a safe level of 2.5 × 44 = 110 µg TTX equivalent/kg shellfish meat. Taking into account this inconclusive situation with regard to the maximum TTX levels that should be considered as safe for shellfish consumption, the EU Aquaculture Advisory Council (AAC) and Market Advisory Council (MAC) have submitted a position paper to the EU, mainly underlining the need for more evidence-based information on TTX toxicity (toxicokinetics, oral toxicity, chronic effects, combination with STX) to reduce the uncertainty factors in a responsible way, before the introduction of a regulatory limit is considered [17].

Further to the need for further data on the acute oral toxicity of TTX, the EFSA opinion also recognized the possibility that, due to their similar modes of action, toxicities of STX and TTX were additive, so they could perhaps be combined to yield one health-based guideline value. In this context, a study was conducted by Finch et al. [65] to determine the toxicity of TTX by a number of different routes of exposure, including feeding, and at the same time to investigate whether the toxicities of STX and TTX are additive. NOAELs for indivicual TTX and STX by feeding were also determined to allow for a better estimation of the ARfD. The testing of three different mixtures of STX and TTX (1:2, 1:1 & 2:1) and subsequent comparison of the experimentally determined values to those predicted on the basis of additive toxicity, demonstrated that the toxicities of STX and TTX are indeed additive. Furthermore, the oral toxicity of TTX by feeding was shown to be the same as that of STX, demonstrating that: (a) it is appropriate to treat TTX as a member of the paralytic shellfish group of toxins, (b) STX and its analogues and TTX and its analogues can be combined to yield one HBGV and (c) a molar toxicity equivalence factor of 1.0 for TTX is applicable. Finally, the NOAEL calculated for TTX was 3.2 μg/kg (10.1 nmol/kg), which is almost 13 times higher than the one proposed in the EFSA opinion. Following the rationale of the EFSA panel, based on a large portion size (400 g) and an adult body weight of 70 kg, the 3.2 μg/kg ARfD would yield a figure of 560 μg TTX/kg of shellfish meat, which would not be expected to lead to adverse effects in humans [65].

#### 5.2.2. Chronic Toxicity

An acute oral lethal dose 50 (LD_50_) of 232 μg/kg TTX per body weight (BW), and a NOAEL of 75 μg/kg after monitoring the mice for two hours have been established [19] and adopted by the EFSA opinion, leading to the introduction of the establishment 44 μg TTX equivalents/kg shellfish meat as a safe concentration in fishery products, but until the time it was published there were no data available with regard to chronic or subchronic oral toxicity of TTXs, this being one of the recommendations of the relevant EFSA Panel for further data necessity [16]. In this context, a preliminary evaluation of the in vivo chronic effects of repeated exposure to low oral TTX doses (below the LD_50_), following internationally adopted guidelines, was conducted by Boente-Juncal et al. [18]. TTX was administrated daily to 4-week old Swiss female mice by gavage for a period of 28 days at doses ranging from 25 to 125 μg/kg. The starting point of 25 μg/kg of TTX was selected as it was the lowest sub-lethal dose proven in acute oral studies to not cause adverse symptoms [19], and dosing levels were subsequently increased to 75 μg/kg and 125 μg/kg. Despite the fact that low TTX doses did not have a major effect on food consumption or body weight, the toxin almost completely suppressed urine production over a 24 h period in mice dosed with TTX at 75 μg/kg and 125 μg/kg. Moreover, repeated oral exposure of mice to TTX altered the urinalysis, with the effects being dose-dependent and manifested by darker urine colors and increasing turbidity, together with moderate ketonuria, bilirubinuria and urobilinogenuria after 28 days oral administration of the toxin. Blood biochemistry parameters were also altered, with a subtle increase in LDH and CK levels, however still being within the normal physiological range. It was also shown that daily repeated exposure of mice to TTX at doses of 125 μg/kg led to ultrastructural changes in the kidney and myocardium. Despite the low number of mice surviving the whole 28-day experimentation at the higher TTX doses, these data constitute a useful initial approach to evaluate the potentially harmful effects after repeated in vivo oral exposure to TTXs [18].

## 6. Conclusions

The importance of the public health risks associated with the presence of TTXs in Europe has led to a significant amount of research undertaken during the two years following the adoption of the relevant EFSA scientific opinion. The progress towards the development of validated analytical methods is noteworthy, despite the shortcomings related to availability of certified standards. On the other hand, most of the EFSA recommendations still need further work to be addressed, with a special focus on the requirement for more data on the individual toxicity of TTX analogues, in order to provide more accurate TEFs, and on the absorption and metabolism of these toxins in both laboratory animals and humans. Additionally, more studies on the sub-acute and chronic toxicity of TTXs, alone or in combination with PSPs, are needed to improve our knowledge on their potential effects on human health, so that the existing risk assessment is refined. The latter could result in the introduction of a legislative maximum acceptable level in shellfish, and/or at least in a uniform regulatory management approach for this toxin group at EU level. It should be noted, however, that Japan is currently the only country with an official regulatory limit for TTXs set at 10 MU/g, equivalent to 2 mg TTX/kg pufferfish tissue [66,67,68], and that this limit has been derived by human epidemiological data, being unchanged through the years due to the fact that no human poisoning cases have occurred below this value. In this context, it is necessary that any attempt to set a maximum acceptable level in shellfish should be cautious and based on solid epidemiological data, as the provisional concentration of 44 μg TTX/kg shellfish meat indicated by the EFSA opinion, may be too low and could jeopardize the viability of the European shellfish industry.

## Figures and Tables

**Table 1:** Recently developed methods for the analysis of TTX and its analogues, described following the approach used in the EFSA opinion [16].

Method Type	Method Principle	Matrix	LOD ^1^	LOQ ^2^	Method Remarks	Reference
**In vitro bioassays**						
Cell-based assay	Sensitivity to neuroblastoma neuro-2a (N2a) cell line	Shellfish	20 µg TTX/kg	n.a. ^3^	Potential false positive results if PSP toxins are present in the sample, even at low levels	[29]
Immunoassay	Competitive inhibition enzymatic immunoassay (mELISA)	Mussels	30 μg TTX/kg	n.a.	No cross-reactivity of the antibody with co-occurring PSP toxins	[30]
		Oysters	20 μg TTX/kg			
	Electrochemical magnetic bead-based immunosensing	Pufferfish tissue	1.2 ng/mL	n.a.	Cross-reactivity with TTX analogues related to concentration and not to toxicity	[31]
Surface Plasmon Resonance (SPR)	Nanoarray planar waveguide biosensor	Pufferfish tissue	0.4 mg/kg	n.a.	Cross-reactivity with some of the known TTX analogues	[32]
**Spectrophotometric methods**						
Spectrofluorimetric	Up-conversion fluorescence using a microfluidic aptasensor	TTX-spiked human biological fluids (gastric juice, human serum and urine)	0.06 ng/mL	1 ng/mL	High detection recoveries (96.0–104.2%), indicating limited interference by other toxins, biomolecules and anions	[33]
**Chemical-analytical methods**						
Liquid Chromatography Mass Spectrometry (LC-MS) - Single analyte group	Hydrophilic Interaction LC - tandem MS (HILIC-MS/MS)	Shellfish (mussels, Pacific oysters)	~0.25 μg/kg	~ 0.8 μg/kg	Matrix-matched calibration. Inclusion of 5 TTX analogues (4-epiTTX; 5,6,11-trideoxyTTX; 11-norTTX-6-ol; 5-deoxyTTX; and 4,9-anhydroTTX). Recovery in mussels: 70%–77% Recovery in oysters: 76%–84%. Expanded uncertainty (k = 2) for TTX: mussels 36% and oysters 51%. LOD, LOQ, and within-laboratory reproducibility satisfactory also for 11-norTTX-6-ol, 5-deoxyTTX, and 4,9-anhydroTTX. Combined acquisition of PSP toxins possible	[34]
	HILIC-MS/MS	Shellfish (mussels, oysters)	Mussels: 0.31 ± 0.12 μg/kg Oysters: 0.36 ± 0.11 μg/kg	Mussels: 1.03 ± 0.34 μg/kg Oysters: 0.86 ± 0.13 μg/kg	Matrix-matched calibration. Inclusion of 6 TTX analogues (4-epiTTX; 5,6,11-trideoxyTTX; 11-norTTX-6-ol; 5-deoxyTTX; 11-deoxyTTX; and 4,9-anhydroTTX). Recovery in mussels: 91.6%–109.5% Recovery in oysters: 69.3%–79.6%	[35]
	Ultra Performance LC-MS/MS (UPLC-MS/MS)	Shellfish (mussels, oysters)	3 μg/kg	20 μg/kg	Matrix-matched calibration. Inclusion of 6 TTX analogues (4-epiTTX; 5,6,11-trideoxyTTX; 11-norTTX-6-ol; 5-deoxyTTX; 11-deoxyTTX; and 4,9-anhydroTTX).	[29]
LC-MS - Multi-analyte toxin group	UPLC-MS/MS	Mussels	0.47 μg/Kg	1.56 μg/Kg	Simultaneous detection and quantification of all EU-regulated marine toxins (both lipophilic and hydrophilic) and TTX (parent and 4,9-anhydroTTX).	[36]
LC-MS - Screening for multiple toxin groups (marine and freshwater)	LC-MS coupled to Fourier transform mass spectrometer (FTMS) - High Resolution LC-MS/MS (LC-HRMS/MS)	Shellfish (mussels, oysters)	n.r. ^4^	n.r.	Broad screening including approx. 800 marine and freshwater toxins, parent TTX and 30 TTX analogues	[29]
LC-MS - Screening and quantitation for TTX and known analogues	LC-HRMS/MS	Puffer fish (*Lagocephalus sceleratus*) shellfish (*Charonia lampas*)	0.041 mg/kg in TTX-spiked mackerel extracts	0.136 mg/kg in TTX-spiked mackerel extracts	Screening for parent TTX and 30 known TTX analogues. Mean recovery (in TTX-spiked mackerel extracts) = 61.17% (57.33–65.00%)	[37]
	LC-HRMS/MS	Puffer fish (*L. sceleratus*)	0.05 mg/kg	0.1 mg/kg	Multi-toxin analysis including TTX all analogues previously described for the species and other analogues described in the literature	[38]
Capillary electrophoresis - mass spectrometry	Capillary electrophoresis coupled to tandem mass spectrometry (CE-MS/MS)	Puffer fish, mussels, sea slug (*Pleurobranchaea maculata*)	Mussels: 0.0052 mg/kg	n.r.	Multiclass analysis of polar marine toxins (paralytic shellfish toxins, tetrodotoxins and domoic acid). Inclusion of parent TTX, 4-epiTTX and 4,9-anhydroTTX (separation evaluated in standard solution)	[39]

^1^ LOD = Limit of detection; ^2^ LOQ = Limit of quantification; ^3^ n.a. = not applicable; ^4^ n.r. = not reported.

**Table 2:** Cumulative TTX testing results for shellfish produced and harvested in the EU, which have not been assessed in the EFSA TTX opinion [16], reported by different countries using LC-MS/MS analysis.

Country	Year(s) of Sampling	Shellfish Species	Samples Tested (*n*) ^1^	TTX-Positive Samples (*n*) ^2^	Maximum TTXs Concentration (μg/kg)	Reference
Italy	2017	Mussels (*Mytilus galloprovincialis*)	2	1	541	[47]
		Wedge clams (*Donax trunculus*)	1	0	n.d. ^3^	
	2018	Mussels (*Mytilus galloprovincialis*)	n.r. ^4^	2	216	[48]
	2015–2017	Mussels (*Mytilus galloprovincialis*)	23	14	6.4	[46]
		Clams (*Venerupis decussata*)	2	0	n.d.	
Netherlands	2015–October 2018	Mussels (*Mytilus edulis*)	1085	38	101	[29,49]
		Oysters (*Crassostrea gigas*)	246	52	253	
		Razor clams (*Ensis* spp.)	113	0	n.d.	
		Cockles (*Cerastoderma edule*)	91	0	n.d.	
Spain	2017	Mussels ^5^	258	0	n.d.	[50]
		Oysters ^5^	28 ^6^	1	0.9	
		Cockles ^5^		1	2.3	
	2018	Mussels, Oysters, Cockles ^5^	72 ^6^	0	n.d.	[51]
France	2018	Mussels ^5^	108	0	n.d.	[52]
		Oysters ^5^	24	0	n.d.	
Ireland	2012–2016	14 shellfish species, including gastropods and grazers ^5^	508 ^6^	0 ^7^	n.d.	[53,54,55,56]
UK	2014–2016	Mussels (*Mytilus edulis*)	1222 ^6^	57 ^8^	73	[22]
		Pacific oysters (*Crassostrea gigas*)			253	
		Native oysters (*Ostrea edulis*)			~ 80	
		Cockles (*Cerastoderma edule*)			n.d.	
		Hard clams (*Mercenaria mercenaria*)			72	
		Surf clams (*Spisula solida*)			n.d.	
		King scallops (whole) (*Pecten maximus*)			n.d.	
		King scallops (adductor & roe) (*Pecten maximus*)			n.d.	

^1^ n = number of samples; ^2^ TTX-positive = above method Limit of Detection (LOD), unless otherwise indicated; ^3^ n.d. = not detected; ^4^ n.r. = not reported; ^5^ Individual numbers of samples by species not reported; ^6^ Species scientific name(s) not reported; ^7^ TTX-positive = above method Limit of Quantification (LOQ) = 40 μg/kg; ^8^ TTX-positive = above method Limit of Reporting (LOR) = 2 μg/kg.

**Table 3:** Number of samples and TTXs contents (μg/kg) in shellfish produced and harvested in the Netherlands from 2015 to October 2018 analyzed by LC-MS/MS (adapted from [29] and 49]).

Year	Parameter	Mussels	Oysters	Razor Clams	Cockles	Total
2015	n ^1^	183	41	20	13	257
	C > LOD ^2^	7	8	0	0	15
	C > LOQ ^3^	n.r. ^4^	n.r.	0	0	5
	Max. value	n.r.	124	n.a. ^5^	n.a.	124
2016	n	280	60	38	25	403
	>LOD (n/%)	17	19	0	0	36
	>LOQ (n/%)	n.r.	n.r.	0	0	20
	Max. value	101	253	n.a.	n.a.	253
2017	n	281	61	36	25	403
	>LOD (n/%)	8	10	0	0	18
	>LOQ (n/%)	3	3	0	0	6
	Max. value	n.r.	51	n.a.	n.a.	51
2018	n	341	84	19	28	472
	>LOD (n/%)	3	10	0	0	13
	>LOQ (n/%)	0	2	0	0	2
	Max. value	n.a.	54	n.a.	n.a.	54

^1^ n = number of samples analyzed. ^2^ Method LOD (Limit of detection) = 3 μg TTX/kg. ^3^ Method LOQ (Limit of quantification) = 20 μg TTX/kg. ^4^ n.r. = not reported. ^5^ n.a. = not applicable.
